# Expression and function of Ndel1 during the differentiation of neural stem cells induced by hippocampal exosomesticle

**DOI:** 10.1186/s13287-020-02119-2

**Published:** 2021-01-09

**Authors:** Wen Li, Shanshan Wang, Hui He, Jianbing Qin, Xiang Cheng, Heyan Zhao, Meiling Tian, Xinhua Zhang, Guohua Jin

**Affiliations:** 1 Department of Human Anatomy, Institute of Neurobiology, Medical School of Nantong University, No. 19 Qixiu Road, No. 3 Building of Qixiu Campus, Nantong, 226001 Jiangsu China; 2 Key Laboratory of Neuroregeneration of Jiangsu and Ministry of Education, No. 19 Qixiu Road, No.3 Building of Qixiu Campus, Nantong, 226001 Jiangsu China; 3 Co-Innovation Center of Neuroregeneration, Medical School of Nantong University, No.19 Qixiu Road, No. 3 Building of Qixiu Campus, Nantong, 226001 Jiangsu China; 4 Department of Anatomy and Neurobiology, Jiangsu Key Laboratory of Neuroregeneration, Collaborative Innovation Center of Neuroregeneration, Medical School of Nantong University, Nantong, Jiangsu China

**Keywords:** Exosomes, Ndel1, miR-107-3p, Neural stem cells, Neurons, Hippocampus

## Abstract

**Background:**

In the brain of adult mammals, neural stem cells persist in the subventricular zone of the lateral ventricle and the subgranular zone of the dentate gyrus, which are specialized niches with proliferative capacity. Most neural stem cells are in a quiescent state, but in response to extrinsic stimuli, they can exit from quiescence and become reactivated to produce new neurons, so neural stem cells are considered to be a potential source for cell replacement therapy of many nervous system diseases. We characterized the expression of Ndel1 during the differentiation of neural stem cells induced by hippocampus exosomes, and assessed the effect of Ndel1 on neural stem cells differentiation.

**Methods:**

Hippocampal exosomes were isolated and extracted, and co-cultured exosomes with neural stem cells. Western blot, flow cytometry, and immunofluorescence analyses were used to analyze expression of neuronal markers. Further, utilizing high-throughput RNA sequencing technology, we found that nudE neurodevelopment protein 1-like 1 was significantly upregulated in exosomes derived from denervated hippocampus, and then characterized its mechanism and function during neural stem cells differentiation by qRT-PCR, western blot, flow cytometry, and immunofluorescence analyses.

**Results:**

Our results revealed that exosomes of denervated hippocampus promoted the differentiation of neural stem cells into neuron. Hence, we identified that nudE neurodevelopment protein 1-like 1 was significantly upregulated and highly expressed in the nervous system. In addition, we found that miR-107-3p may regulate neural stem cell differentiation by targeting Ndel1.

**Conclusions:**

Our results revealed that deafferentation of the hippocampal exosomes co-cultured with neural stem cells could promote them to differentiate into neurons. Hence, we found that miR-107-3p may regulate neural stem cells differentiation by targeting Ndel1. Importantly, Ndel1 enhanced spatial learning and hippocampal neurogenesis in rats after fimbria fornix transection in vivo. These findings set the stage for a better understanding of neurogenesis, a process that 1 day may inspire new treatments for central nervous system diseases.

## Introduction

The hippocampus originates from the medial pallium of the dorsal telencephalon and plays important roles in learning, memory, and affective behaviors [32]. The subgranular zone of the hippocampal dentate gyrus (DG) is one of the stem-cell-containing niches in the adult mammalian brain [1]. This thin band between the granule cell layer and the hilus provides a unique microenvironment for the adult neural stem cells (NSCs) population [7]. Heterogeneous pools of NSCs in the adult mammalian brain are the source of new neurons that contribute to brain maintenance and regeneration [17]. Most adult NSCs are quiescent and show a low metabolic rate and a high sensitivity to their microenvironment [29]. The balance of NSC activation and quiescence, as well as the induction of lineage-specific transcription factors, may contribute to the generation of neuronal or glial progeny cells [8].

Exosomes are nano-sized extracellular vesicles secreted by a variety of cell types that have been proven to be important intercellular messengers and exhibit molecular profiles that reflect normal and disease states [19]. A recent study revealed that exosomes in the brain can play critical roles in central nervous system (CNS) diseases, such as stroke [23], Alzheimer’s disease (AD) [19], Parkinson’s disease (PD) [22], prion disease [3], amyotrophic lateral sclerosis (ALS) [27], Huntington’s disease (HD) [15], and chronic traumatic encephalopathy (CTE) [20], with both positive and negative effects. As key mediators of cell-to-cell and distant communication, exosomes are involved in various biological processes, potentially through transferring their contents including proteins, lipids, and RNAs to target cells [5].

Our previous research showed that the deafferent hippocampus provided a supportive microenvironment for the survival, migration, and neuronal differentiation of endogenous hippocampal and implanted NSCs. Importantly, extracts from the denervated hippocampus promoted more NSCs to differentiate into neurons and their subsequent in vitro maturation [33, 34]. These results indicated that deafferentation led to changes in the hippocampal expression of molecules that regulated NSC differentiation. However, it remains unknown whether deafferentation of the hippocampal exosomes could promote the differentiation of NSCs. Our results revealed that deafferentation of hippocampal exosomes co-cultured with NSCs could promote neuronal differentiation. Subsequently, we found that nuclear distribution protein like 1 (Ndel1) was significantly upregulated and highly expressed in the nervous system. Additionally, we found that Ndel1 enhanced spatial learning and hippocampal neurogenesis in rats after fimbria fornix (FF) transection in vivo. These findings revealed a novel mechanism and identified specific targets for treating CNS diseases.

## Materials and methods

### Animals and surgery

Pregnant Sprague-Dawley rats, 1-day-old neonatal Sprague-Dawley rats, and adult Sprague-Dawley rats (weighing 220–250 g) were obtained from the Experimental Animal Center of Nantong University (Certificate No: SYXK (SU) 2012-0031). All experimental procedures were approved by the local Animal Care Committee and were conducted in accordance with the guidelines of the National Institutes of Health (NIH) on animal care and with other relevant the ethical guidelines.

FF transections were performed as described by Hefti [10]. Briefly, after chlorpent anesthesia (2 mL/kg body weight, intraperitoneal), adult SD rats were transferred to the stereotaxic apparatus, and then, FF transection was performed with a wire knife at the CA1 layer of the dorsal hippocampus, at coordinates of bregma: AP = 1.4, ML = 1.0 and AP = 1.4, ML = 4.0, and depth 5.6 mm. There were no restrictions on the sex of the experimental animals.

### Exosome isolation

Seven days following FF transection, deafferented and normal hippocampi were quickly dissected, trypsinized, and homogenized into ice-cold phosphate-buffered saline (PBS). Exosomes were precipitated using Total Exosome Isolation reagent (Invitrogen, Carlsbad, CA, USA) according to the manufacturer’s instructions. Homogenates were centrifuged at 2000×*g* at 4 °C for 30 min to remove cells and debris, and then, supernatants were passed through a 0.22-μm filter to remove extracellular vesicles larger than exosomes. The supernatants were transferred to a new tube without disturbing the pellet and mixed with 0.5 volumes of Total Exosome Isolation reagent and incubated overnight at 4 °C. The mixture was then centrifuged at 10,000×*g* for 30 min, and the supernatant was decanted, while the exosome pellet was resuspended into 100 μL PBS.

### Cell culture

The isolation, culture, and differentiation of NSCs were performed as previously described with some modifications [9]. Briefly, pregnant SD rats were anesthetized, and the embryos were removed by cesarean section. Hippocampi were dissected from embryonic day 14.5 (E14.5) embryos and were then mechanically dissociated into a single-cell suspension. After centrifugation and resuspension, the cell suspensions were plated into flasks with a 1:1 Dulbecco’s modified Eagle’s medium (DMEM) and Ham F-12 mixture (both, Gibco, Grand Island, NY, USA) containing 2% B27 (Gibco), 20 ng/mL epidermal growth factor (EGF; Sigma-Aldrich, St. Louis, MO, USA), and 20 ng/mL basic fibroblast growth factor 2 (bFGF; Sigma-Aldrich). Cells were passaged every 6 days to obtain neurospheres that originated from a single primary cell. For in vitro differentiation, cell suspensions were plated with DMEM/F-12 medium supplemented with 2% B27 and 2% fetal bovine serum (FBS, Gibco). For the mixed co-culture experiments, isolated exosomes were mixed with NSCs and processed in different ways after cocultivation.

Primary neurons were isolated using standard methods, as previously described [31]. Briefly, hippocampi were dissected from E14.5 embryos, and the resultant single cell suspensions were diluted in serum-free neurobasal medium (Gibco) containing 2% B27 and 0.5 mM l-glutamine (Gibco). The cells were then seeded onto plates precoated with poly-d-lysine. Half of the medium was replaced every 3 days.

Primary astrocytes were derived from cerebral cortices of 1-day-old neonatal rats as previously described [31]. Briefly, dissociated cortical cells were suspended in DMEM/F-12 containing 10% FBS and plated in flasks. After 3–4 days, the heterogeneous primary cells were orbitally shaken to remove microglia and oligodendrocytes. Astrocytes were dissociated by trypsinization and then replated into flasks.

### Transfection, lentiviral transduction, and injection

Prior to transfection or transduction, cells were cultured in plates overnight. Cells were transfected with the miR-107-3p/NC mimic or miR-107-3p/NC inhibitor (Ribobio, Guangzhou, China) using Lipofectamine 3000 (Invitrogen) according to the manufacturer’s instructions. Cells were transduced with lentivirus that was constructed by GeneChem Company (Shanghai, China), including overexpression lentivirus (abbreviated as LV-Ndel1) and interference lentivirus (abbreviated as LV-Ndel1i), corresponding to the negative control lentiviruses (LV-NC and LV-NCi) following the manufacturer’s instructions. Green fluorescence expression was then observed under a fluorescence microscope (Axio Scope A1, Zeiss, Oberkochen, Germany). The cells were cultured with lentivirus for 12 h to obtain the best infection complex value, after which the lentivirus was removed and replaced with fresh medium.

In total, 60 SD rats were used for lentivirus injections into the hippocampus. Briefly, after chlorpent anesthesia, adult SD rats were transferred to the stereotaxic apparatus. On day 7 after FF transection, injections of virus into the left and right hippocampal DG at two points were performed at the following coordinates: 3.6 mm to bregma, 1.39 mm to the right or left of the midline, and 3.9 mm in depth. Five microliters of virus was loaded into an internal cannula needle with cannula tubing connected to a Hamilton syringe mounted onto a microinjection pump (Harvard Apparatus, Dover, MA, USA). The speed of the injection was 0.5 μL/min. The needle was kept in position for an additional 10 min after completing the injection and then was slowly retrieved from the brain.

### RNA preparation and qRT-PCR

Isolation of total RNA from tissues and cells was performed using TRIzol reagent (Vazyme Biotech, Nanjing, China) according to manufacturer’s instructions. For mRNA expression analysis, 1 μg of RNA was reverse transcribed into cDNA using the HiScript Q RT SuperMix for qPCR (+gDNA wiper) Kit (Vazyme Biotech). The SYBR green (Roche, Basel, Switzerland) method was performed using a StepOnePlus RealTime PCR system (Applied Biosystems, Waltham, MA, USA) according to the manufacturer’s instructions. The sequences of primers used for qRT-PCR are displayed in Table S1.

For miRNA expression analysis, the miRcute Plus miRNA First-Strand cDNA Synthesis Kit (Tiangen Biotech, Beijing, China) and the miRcute miRNA qPCR Detection Kit (SYBR Green; Tiagen Biotech) were used. According to the manufacturer’s protocol, 1 μg of total RNA was used. Forward primers for miRNAs were obtained from Ribobio (Guangzhou, China), and the reverse primer was commercially available and supplied in the miRcute miRNA qPCR Detection Kit. The 2^−ΔΔCT^ method was used to calculate expression levels from qPCR data.

### Western blot analysis

Briefly, proteins were extracted, quantified, isolated by 10% SDS-PAGE, transferred to 0.2 mm polyvinylidene fluoride membranes, and then blocked with 5% skim milk for 2 h. After incubating with primary antibodies overnight at 4 °C, the membranes were incubated with HRP-linked secondary antibodies for 2 h. Immunoreactive bands were viewed by enhanced chemiluminescence reagents (Bio-Rad, Hercules, CA, USA). The primary antibodies used included anti-Tuj1 (1:1000; Millipore, Billerica, MA), anti-MAP2 (1:1000; Abcam, Cambridge, UK), anti-Ndel1 (1:1000; Abcam), and anti-β-actin (1:1000; Abcam).

### Immunofluorescence and immunohistochemistry

Cells and tissues were fixed with 4% paraformaldehyde for 30 min, washed with PBS three times, permeabilized and blocked with 10% normal goat serum containing 0.3% Triton X-100 and 1% BSA for 2 h, and then incubated with primary antibody overnight at 4 °C. For immunofluorescence, cells and tissues were washed three times with PBS and incubated with the corresponding fluorescent secondary antibody at room temperature for 2 h. Nuclei were counterstained with Hoechst 33342 (1:1000; Pierce, Rockford, IL, USA). Primary antibodies included anti-Tuj1 (1:1000; Millipore) and anti-MAP2 (1:1000; Abcam). Images were captured by using a fluorescence microscope.

Immunohistochemistry was performed using a Super-Sensitive Horseradish Peroxidase Immunohistochemistry Kit (rabbit; Sangon Biotech, Shanghai, China). Sections were incubated with rabbit anti-Ndel1 antibody (1:1000, Abcam) at 4 °C overnight followed by incubation with poly-HRP-conjugated anti-rabbit IgG. After rinsing in PBS, sections were detected using a DAB working solution.

### Flow cytometry

Cells were fixed in a 1× Fix/Perm Buffer working solution at 4 °C for 40 min. After washing with 1× Perm/Wash Buffer, the cell samples were mixed with 80–100 μL of 1× Perm/Wash Buffer and incubated with APC-conjugated anti-Tuj1 antibody or APC-conjugated IgG2A Control (BD Biosciences) (Figure S1A) at 4 °C for 2 h. Cells were centrifuged and resuspended in flow cytometry stain buffer and then analyzed using a flow cytometer.

### Luciferase reporter assay

The luciferase reporter vectors were constructed by GeneChem. For the luciferase reporter assays, HEK-293 cells plated in a 24-well plate were co-transfected with 100 ng plasmid and 100 ng luciferase construct. Luciferase and Renilla signals were measured 72 h after transfection using the Dual-Luciferase Reporter Assay Kit (Promega, Madison, WI, USA) according to a protocol provided by the manufacturer.

### Statistical analysis

Statistical analyses were mainly conducted using GraphPad Prism 6.0 (GraphPad Software Inc., San Diego, CA, USA). Differences between two groups were compared using an unpaired Student’s two-tailed *t* test, and differences among multiple groups were analyzed by one-way ANOVA. The results were considered statistically significant when *P< 0.05, **P< 0.01, and ***P< 0.001.

## Results

### Effects of hippocampal exosomes on NSC differentiation

**Fig. 1:**
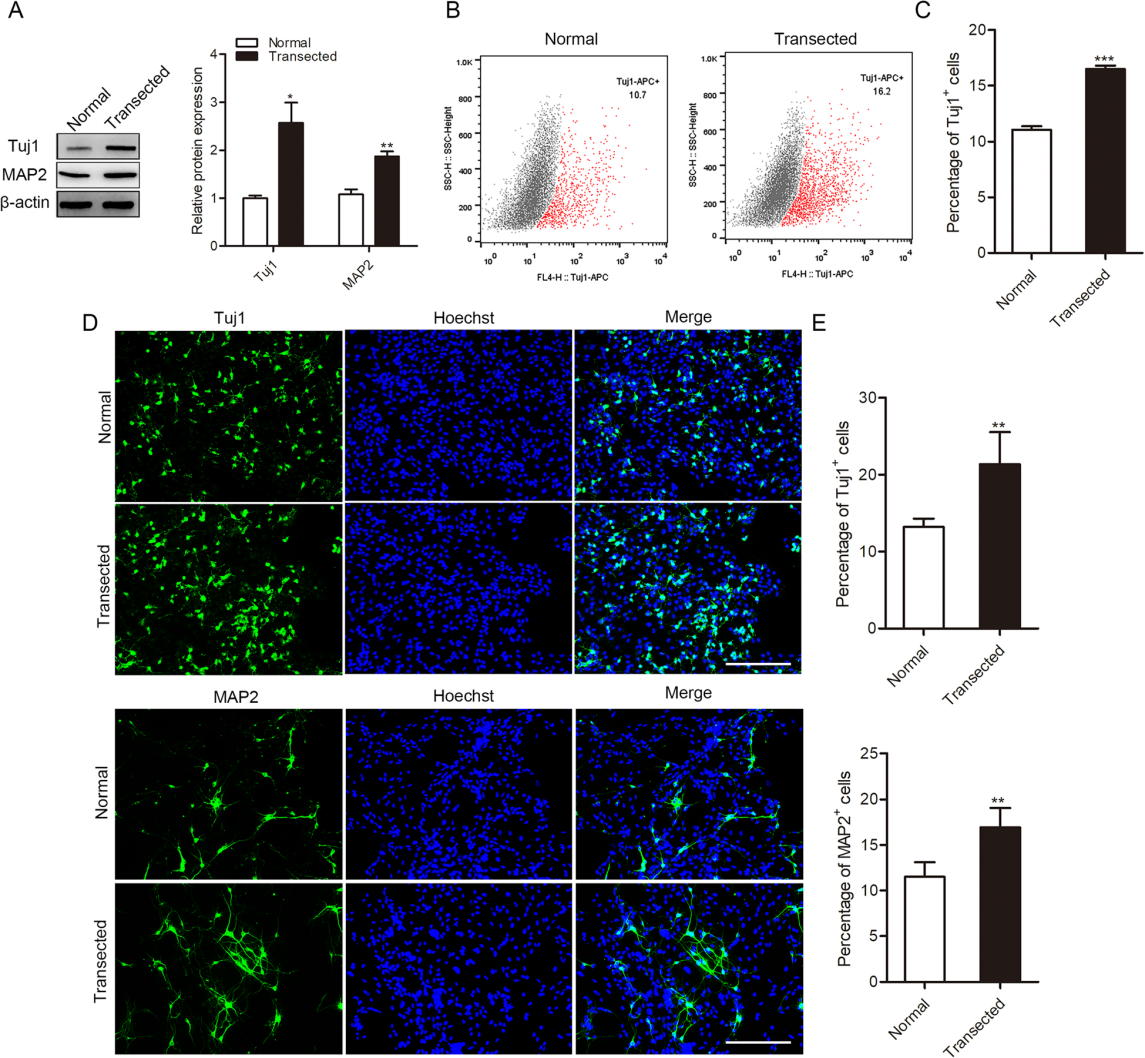
Effects of hippocampal-derived exosomes on NSC differentiation. **a** Western blot analysis of Tuj1 and MAP2 protein levels. (normal) NSCs treated with normal hippocampal exosomes; (transected) NSCs treated with deafferented hippocampal exosomes. **b**, **c** The percentage of Tuj1-positive cells detected by flow cytometry. **d**, **e** Immunofluorescence analysis of Tuj1 (green) and MAP2 (green) cells in the transected and normal group. Nuclei were stained with Hoechst. Scale bar = 200 μm. Values are mean ± SEM from three biological replicates; *P < 0.05, **P < 0.01, ***P < 0.001

To identify the isolated exosomes, we applied transmission electron microscopy. As shown in Figure S1B, hippocampal-derived exosomes were lightly stained and had diameters within 30**~**200 nm. To confirm that these exosomes could be transferred to cells, we co-cultured CM-Dil-labeled exosomes with NSCs. After incubation with exosomes, the CM-Dil fluorescence signal was observed in most NSCs (Figure S1C). As shown in Fig. 1a, Western blotting showed that Tuj1 and MAP2 were significantly upregulated in the transected group. Similarly, flow cytometric analysis showed that there were more Tuj1-positive cells in the transected group than in control (Fig. 1b, c). Immunofluorescence staining showed that percentage Tuj1- and MAP2-positive cells were upregulated (Fig. 1d, e). Our results also revealed that exosomes derived from deafferented hippocampi facilitated neuronal differentiation of NSCs.

### High-throughput functional screening for differentially expressed mRNAs

**Fig. 2:**
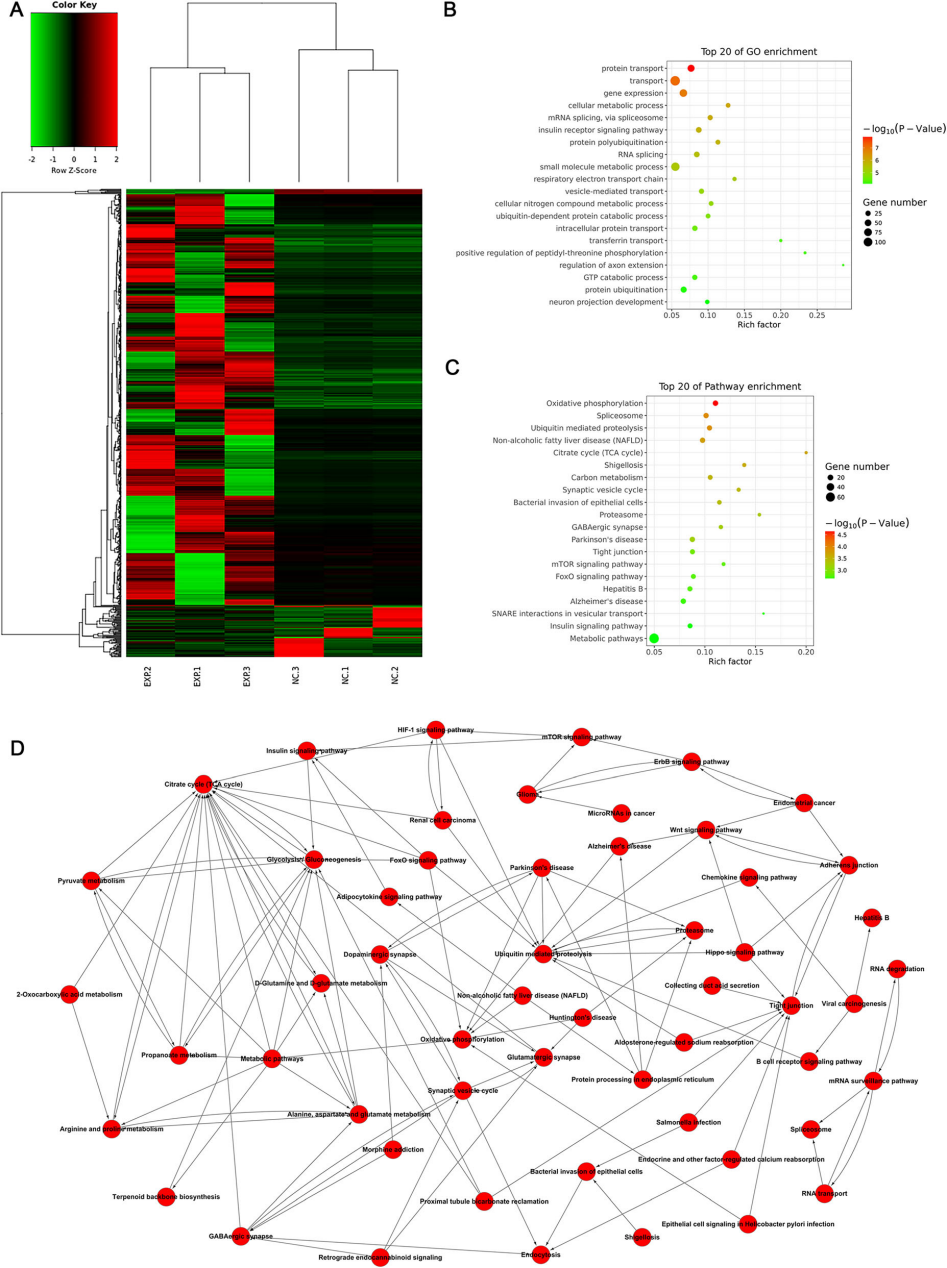
Differentially expressed mRNAs in hippocampal-derived exosomes. **a** Clustered heatmap of differentially expressed mRNAs. **b** GO analysis of differentially expressed mRNAs. **c** KEGG pathway analyses of differentially expressed mRNAs. **d** The relationships between enriched pathways, where red represents upregulated pathways in exosomes

To identify and characterize the differentially expressed exosomal mRNAs, RNA-seq was implemented in three pairs of hippocampal exosomes. When we set the filter criteria to be fold-change ≥ 2 and a *p* value < 0.05, we found 770 differentially expressed mRNAs, among which 764 were upregulated and six were downregulated in hippocampal exosomes (Table S2). The heat map of differentially expressed genes is shown in Fig. 2a. Next, a bioinformatics analysis was performed to characterize the mRNA profile of hippocampal exosomes. Gene ontology (GO) analyses suggested the differentially expressed genes were associated with protein transport, gene expression, cellular metabolic processes, and other important functions (Fig. 2b). Pathway analyses suggested that oxidative phosphorylation, spliceosome, and ubiquitin-mediated proteolysis were most enriched among the differentially expressed genes (Fig. 2c). Figure 2d presents the relationships between enriched pathways.

### Identification and characteristics of Ndel1

**Fig. 3:**
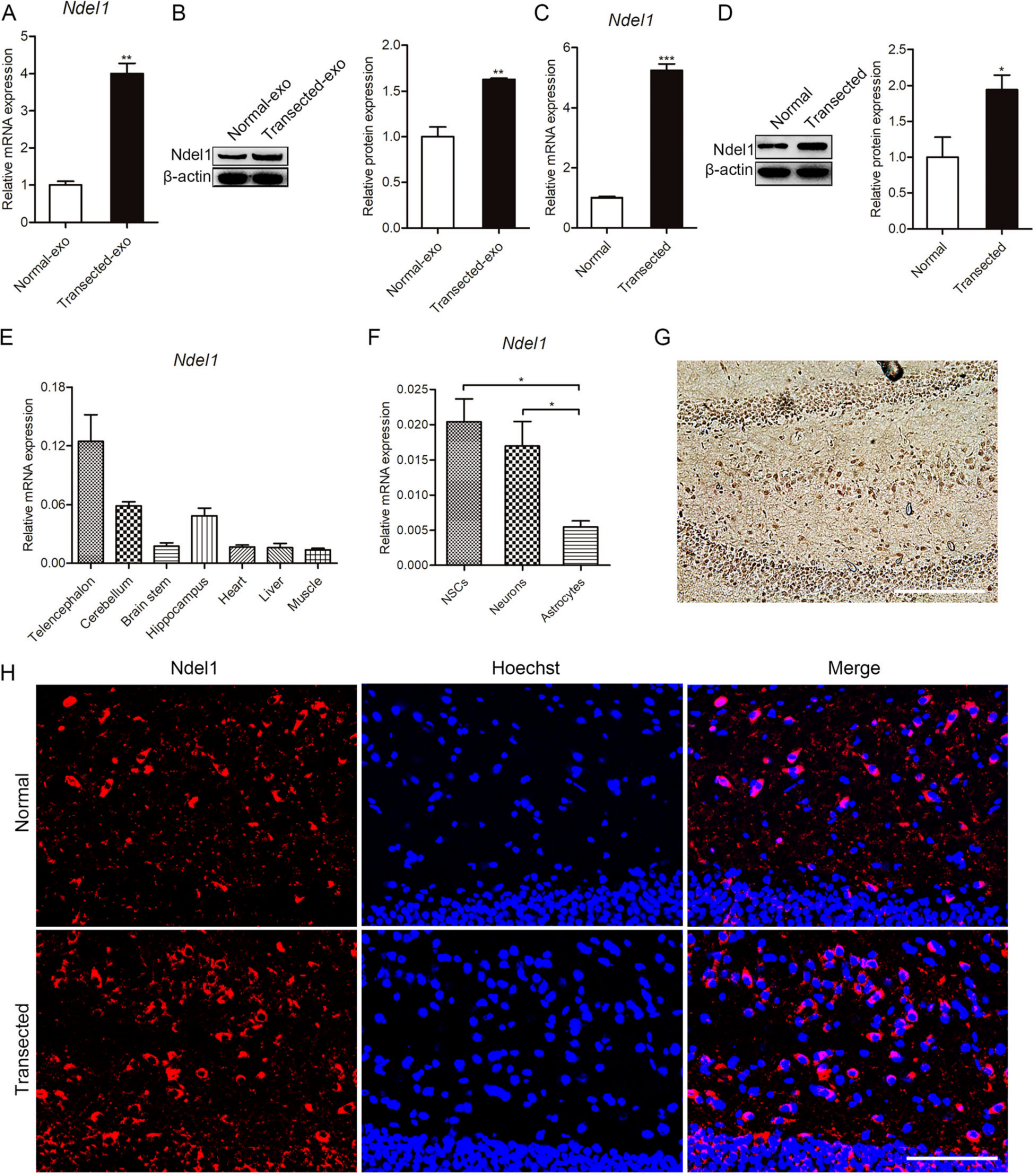
Identification and characteristics of Ndel1. **a**, **b** RT-qPCR and Western blot analysis confirming the changes of Ndel1 in exosomes. (normal-exo) exosomes of normal hippocampi; (transected-exo) exosomes of hippocampi after fimbria fornix (FF) transection. **c**, **d** Ndel1 expression measured by RT-qPCR and Western blots after incubation. **e** Analysis of Ndel1 expression in different tissues. **f** Ndel1 expression in NSCs, neurons and astrocytes. **g** Immunohistochemical staining showing the distribution of Ndel1 in the adult rat hippocampus. **h** Immunofluorescence staining showing Ndel1 expression in the adult rat hippocampus after FF transection. Nuclei were stained with Hoechst. Scale bar = 200 μm. Values are mean ± SEM from three biological replicates; ^*^P < 0.05, ^**^P < 0.01, ^***^P < 0.001

Among the upregulated mRNAs, we focused on Ndel1, which was enriched in neuron projection development, microtubule cytoskeleton organization, nervous system development, and central nervous system neuron axonogenesis according to GO analysis. As shown in Fig. 3a, b, differential expression of exosomal Ndel1 was consistent with the trends observed using RNA sequencing. Furthermore, after being co-cultured with NSCs, we found that Ndel1 expression was increased in the transected group (Fig. 3c, d). To explore the Ndel1 expression pattern, we extracted RNA from tissues derived from the ectoderm (cerebrum, cerebellum, brain stem, and hippocampus), mesoderm (heart and muscle), and endoderm (liver) and then performed a RT-qPCR analysis. As shown in Fig. 3e, Ndel1 was significantly overexpressed in the nervous tissues compared with other tissues. Additionally, Ndel1 showed its highest expression in NSCs, followed by neurons, and minimally in astrocytes (Fig. 3f). We then examined the expression pattern of Ndel1 in the hippocampus by immunohistochemistry. The results showed that Ndel1 was more highly localized to the somata of some polymorph layer cells, but was also expressed in the granular layer of the DG (Fig. 3g). Seven days after FF injury, we found that the number of Ndel1-positive cells had increased in the denervated hippocampus (Fig. 3h). These data suggested that Ndel1 played an important role in neurogenesis.

### Effects of Ndel1 on NSC differentiation

**Fig. 4:**
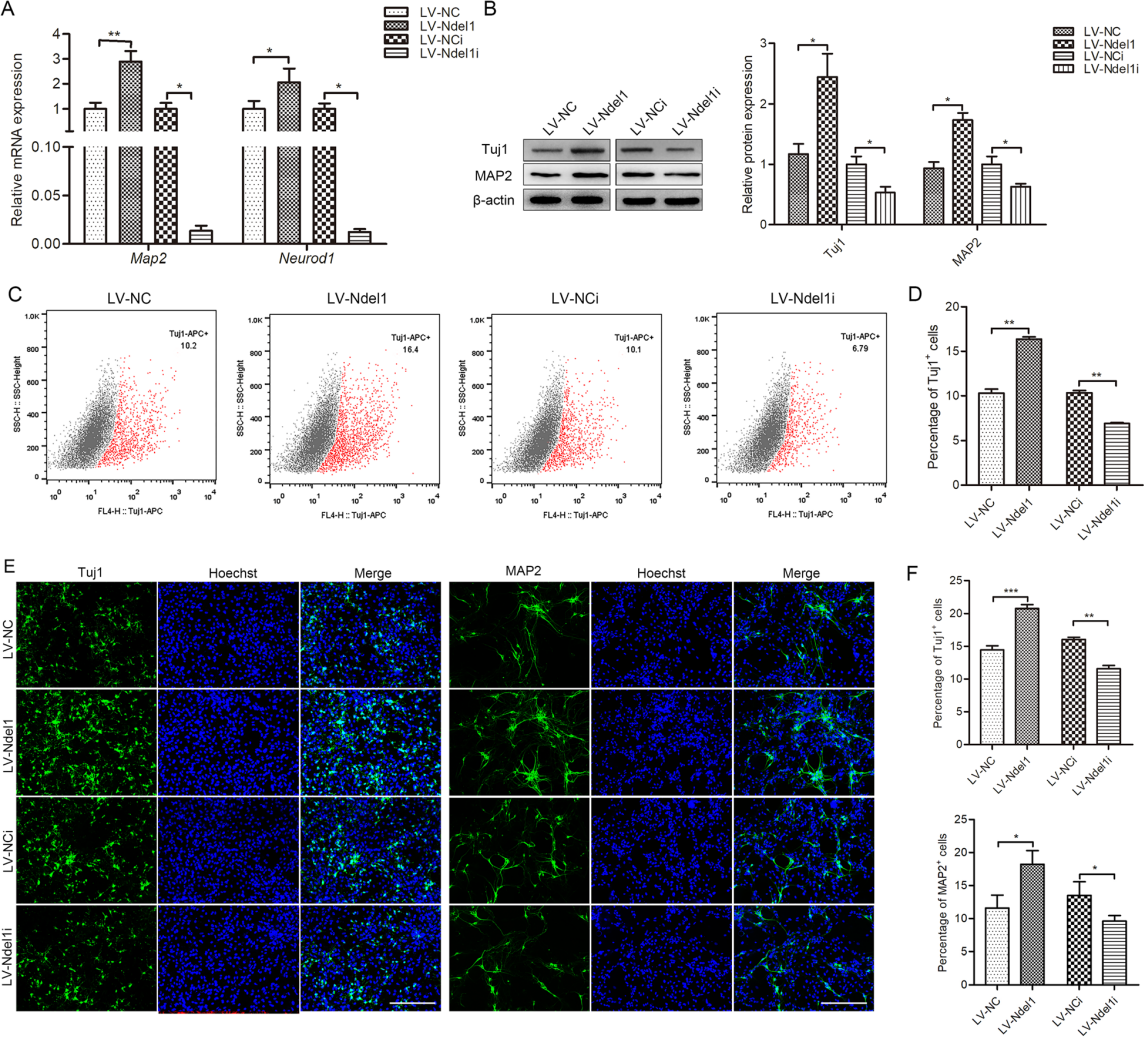
Effects of Ndel1 on NSC differentiation. **a** RT-qPCR analysis of *Map2* and *Neurod1* expression. (LV-NC) NSCs treated with negative control of overexpression lentivirus; (LV-Ndel1) NSCs treated with overexpression lentivirus of Ndel1; (LV-NCi) NSCs treated with negative control of interference lentivirus; (LV-Ndel1i) NSCs treated with interference lentivirus of Ndel1. **b** Western blot analysis of Tuj1 and MAP2 protein levels. **c**, **d** The percentage of Tuj1-positive cells detected by flow cytometry. **e**, **f** Cell differentiation was detected by immunofluorescence. Nuclei were stained with Hoechst. Scale bar = 200 μm. Values are mean ± SEM from three biological replicates; *P < 0.05, **P < 0.01, ***P < 0.001

To examine the precise functions of Ndel1 in NSCs, we transfected NSCs with lentiviral vectors encoding Ndel1 (Figure S1D-S1G). To explore whether Ndel1 regulated NSC differentiation, we measured the expression levels of two commonly used nerve-specific molecules, *Map2* and *Neurod1*. The results showed that Ndel1 upregulation promoted *Map2* and *Neurod1* expression. Knocking down Ndel1 had the opposite effect (Fig. 4a). Western blotting showed that overexpressing Ndel1 notably increased Tuj1 and MAP2 expression. Conversely, knocking down Ndel1 induced decreased Tuj1 and MAP2 expression (Fig. 4b). Consistent with these results, flow cytometry and immunofluorescence revealed that overexpressing Ndel1 notably increased the number of neurons. Conversely, knocking down Ndel1 induced a decrease of neurons. (Fig. 4c–f). Together, these results implied that Ndel1 promoted the neuronal differentiation of NSCs.

### The miR-107-3p suppressed NSC differentiation by targeting Ndel1

**Fig. 5:**
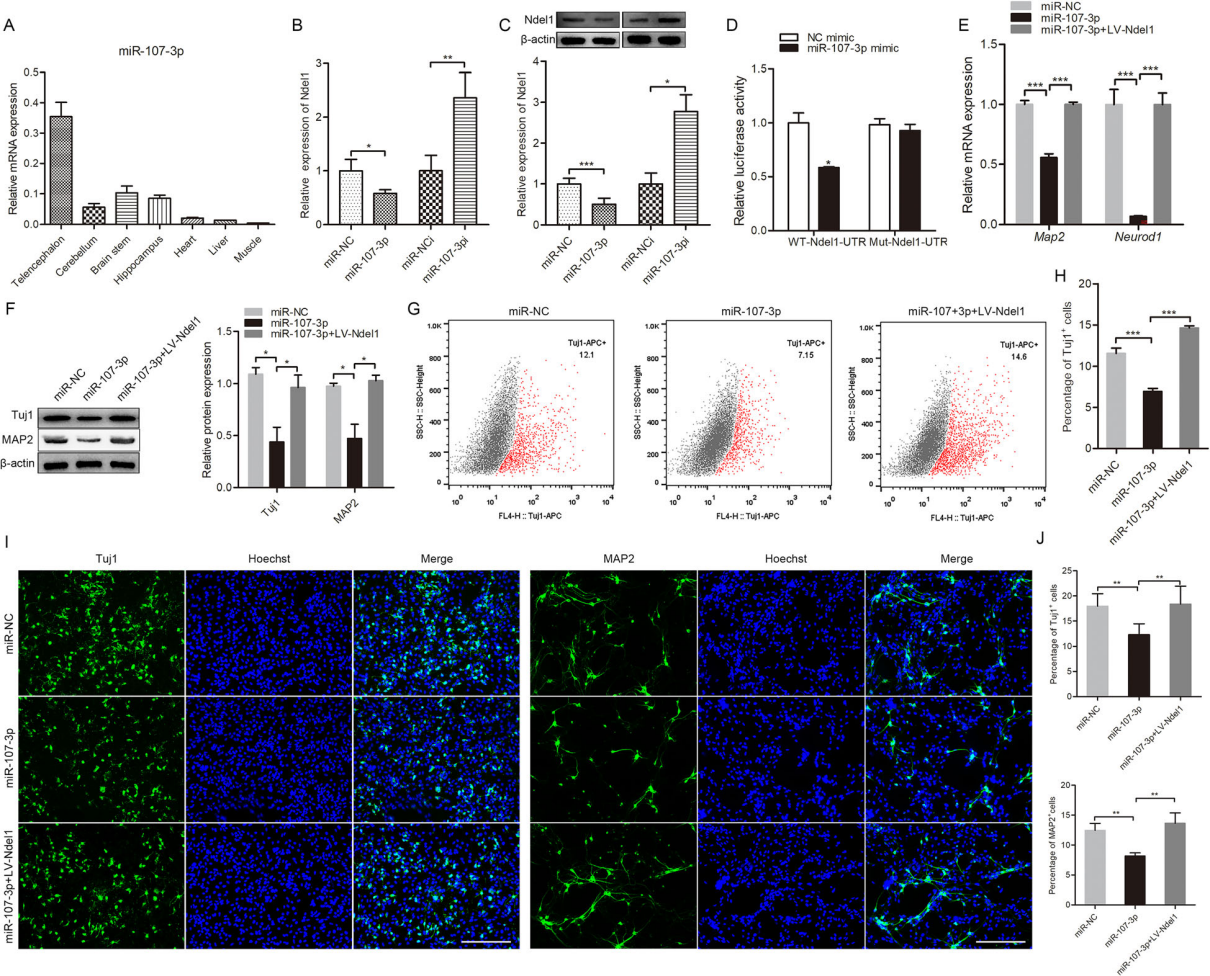
The miR-107-3p regulated NSC differentiation by targeting Ndel1. **a** Analysis of miR-107-3p expression in different tissues. **b**, **c** RT-qPCR and Western blot analysis confirming the changes in Ndel1 expression. (miR-NC) NSCs treated with negative control of miRNA mimic; (miR-107-3p) NSCs treated with miR-107-3p mimic; (miR-NCi) NSCs treated with negative control of miRNA inhibitor; (miR-107-3pi) NSCs treated with miR-107-3p inhibitor. **d** Luciferase reporter assay in HEK-293 cells co-transfected with Luciferase-miR-107-3p fusion and Ndel1-Wild or Ndel1**-**Mut, 72 h post-transfection. **e** RT-qPCR analysis of *Map2* and *Neurod1* expression. (miR-NC) NSCs treated with negative control of miRNA mimic; (miR-107-3p) NSCs treated with miR-107-3p mimic; (miR-107-3p+LV-Ndel1) NSCs treated with miR-107-3p mimic and overexpression lentivirus of Ndel1. **f** Western blot analysis of Tuj1 and MAP2 protein levels. **g**, **h** The percentage of Tuj1-positive cells detected by flow cytometry. **i**, **j** Cell differentiation was detected by immunofluorescence. Nuclei were stained with Hoechst. Scale bar = 200 μm. Values are mean ± SEM from three biological replicates; *P < 0.05, **P < 0.01, ***P < 0.001

To probe the underlying molecular mechanisms of Ndel1, we first used three algorithms (miRWalk, TargetScan, and miRDB) to predict potential upstream miRNA of Ndel1. For all three algorithms, miR-107-3p was the commonly predicted target. We also found that miR-107-3p exhibited high expression in the nervous tissues (Fig. 5a). To further investigate the potential biological function of miR-107-3p, we constructed a miR-107-3p mimic and inhibitor. qRT-PCR results showed that miR-107-3p expression was significantly upregulated and downregulated in NSCs transfected with the miR-107-3p mimic and inhibitor, respectively (Figure S1H). Next, we investigated the impact of miR-107-3p on Ndel1 expression by qRT-PCR and western blot. The results showed that overexpression and knockdown of miR-107-3p resulted in the downregulation and upregulation of Ndel1 in NSCs, respectively (Fig. 5b, c).

Luciferase reporter assays showed that miR-107-3p inhibited the luciferase activity of wild type Ndel1 but not mutant Ndel1 (Fig. 5d). As shown in Fig. 5e, overexpressing miR-107-3p significantly decreased *Map2* and *Neurod1* expression levels, whereas restoring Ndel1 rescued their expression. Similarly, western blotting showed that overexpressing miR-107-3p suppressed Tuj1 and MAP2 expression. Conversely, overexpressing Ndel1 caused increased Tuj1 and MAP2 expression (Fig. 5f). Moreover, flow cytometric analysis and immunofluorescence showed that upregulating miR-107-3p inhibited the neuronal differentiation of NSCs, while increasing Ndel1 expression had the opposite effect (Fig. 5g–j)**.** Together, these results suggested that miR-107-3p suppressed the differentiation of NSCs into neurons by targeting Ndel1.

### Ndel1 enhanced hippocampal neurogenesis in vivo after FF transection

**Fig. 6:**
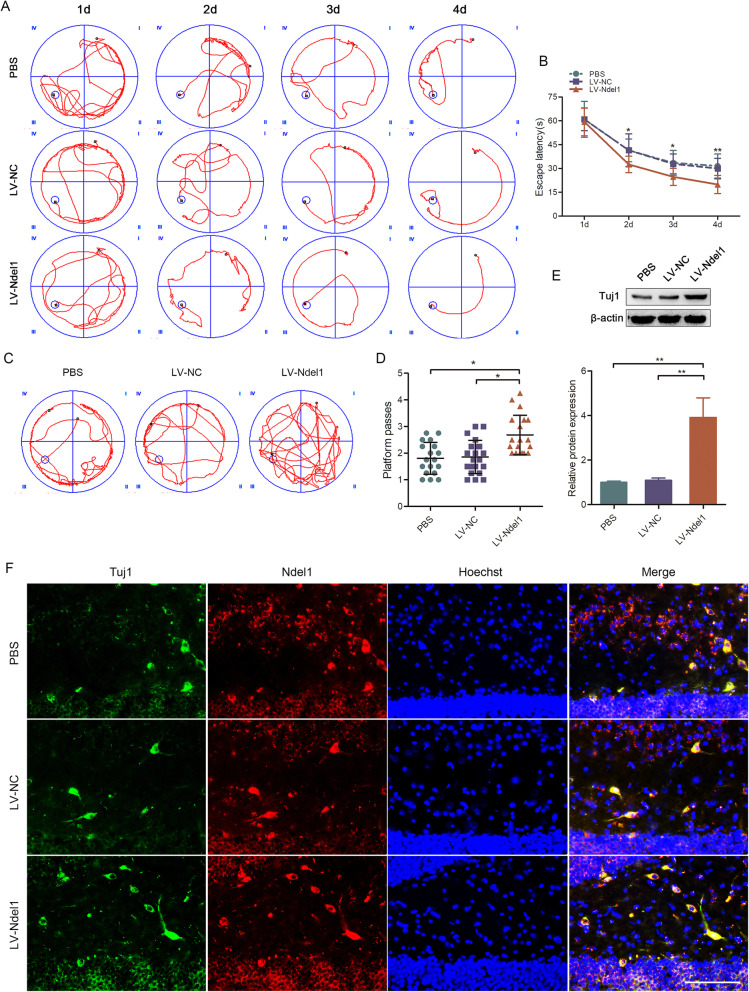
Ndel1 enhanced hippocampal neurogenesis. **a** Representative trajectory diagrams to reach the platform. (PBS) PBS injectioned into the hippocampus; (LV-NC) negative control lentivirus injectioned into the hippocampus; (LV-Ndel1) overexpression lentivirus of Ndel1 injectioned into the hippocampus. **b** The time to reach the platform between the PBS or LV-NC group and the LV-Ndel1 group. **c**, **d** Representative trajectory diagrams and the number of platform location crosses during a single 120 s trial. **e** Tuj1 expression was measured by Western blotting. **f** Immunofluorescence analysis of Tuj1 (green) and Ndel1 (red)-positive cells in the hippocampus. Nuclei were stained with Hoechst. Scale bar = 100 μm. The data are presented as mean ± SEM of three independent experiments; *P < 0.05, **P < 0.01

The Morris water maze test was performed in the last 5 days before sacrifice (35 days post injury) to evaluate spatial learning. Compared with rats in the LV-Ndel1 group, the escape latency of rats in the PBS and LV-NC rats to reach the platform was significantly longer (Fig. 6a, b). Furthermore, LV-Ndel1 group rats crossed the platform more frequently (Fig. 6c, d). GFP detection in the hippocampus proved that the lentivirus successfully infected target tissues (Figure S1I). As shown in Fig. 6e, f, Tuj1 was significantly upregulated. Thus, Ndel1 expression was associated with significantly improved learning and memory ability and enhanced neurogenesis in the hippocampus of adult rats following FF transection.

## Discussion

The discovery of NSCs in the adult brain provides evidence that the CNS may have the potential to repair insults by generating new neurons [37]. NSCs are self-renewing and multipotent cells with the potential to differentiate into neurons, astrocytes, and oligodendrocytes [12]. Adult NSCs continuously generate functional neurons throughout life, and this generation is critical for biological functions [21]. Under certain pathological conditions, the endogenous quiescent NSCs become active and participate in neurogenesis [12]. However, the self-repair process is usually inadequate and transient. Therefore, enhancing endogenous neurogenesis or applying exogenous NSCs has become hot topics.

Exosomes are small vesicular structures that range from 30 to 150 nm in diameter and may carry different types of DNA, RNA, and proteins to transfer information between cells [11, 13]. The cargos of CNS exosomes vary according to the cell of origin as well as the cell’s health, stress, and disease status and can be changed in response to environmental situations [28]. Our research showed that deafferented hippocampal exosomes co-cultured with NSCs could promote neuronal differentiation of the NSCs. Furthermore, using high-throughput RNA sequencing technology, we identified Ndel1 to be significantly upregulated and highly expressed in the nervous system. This suggested that certain RNA species occurred within exosomes and played important roles in neurogenesis.

Ndel1 plays multiple roles in neurodevelopmental processes [30]. Ndel1 is broadly expressed in the brain, including in the majority of cortical neurons [24]. Ndel1 deficiency results in neuronal migration defects, fragmented microtubules, dendritic/synaptic pathologies, and early embryonic lethality [14, 26]. Additionally, Ndel1 plays a critical role in neuronal precursor proliferation and differentiation, neuronal migration, neurite outgrowth, and neuronal positioning during brain development [4, 30]. Here, we demonstrated that Ndel1 promoted the neuronal differentiation of NSCs and improved learning and memory abilities after FF transection.

MiRNAs are a class of small noncoding RNAs that either prevent translation or promote the degradation of specific targets by binding to target sequences usually located in the 3′-UTR [6]. To explore the potential molecular mechanism of Ndel1, we used three algorithms to predict miRNAs that could bind Ndel1, which identified miR-107-3p. There are almost no reports on the relationship between miR-107 and NSC differentiation, and to date, most studies on miR-107 have been related to cancer. A growing body of evidence indicates that aberrant miR-107 expression plays a key role in cancers, including breast cancer [18], gastric cancer [16], cervical cancer [36], hepatocellular carcinoma [2], and non-small cell lung cancer [35]. Prendecki et al. indicated that altered miR-107 levels may be a marker of the neurodegenerative process during the course of AD, which is associated with amyloid β metabolism and excessive cell cycle progression [25]. Our study found that miR-107-3p was highly expressed in nervous tissues; moreover, we found that Ndel1 was directly regulated by miR-107-3p. Subsequently, overexpression of miR-107-3p suppressed Ndel1 expression and inhibited the differentiation of NSCs into neurons.

## Conclusions

Our results revealed that deafferentation of the hippocampal exosomes co-cultured with NSCs could promote them to differentiate into neurons. Hence, we identified that Ndel1 was significantly upregulated and highly expressed in the nervous system. In addition, these results suggested that miR-107-3p may regulate NSC differentiation by targeting Ndel1. With a better understanding of endogenous NSCs under normal and pathological conditions, we may be able to employ endogenous NSCs for neuroregeneration in the future.

## Supplementary Information

**Additional file 1: Figure S1.** Representative images and histograms. (A) The control of flow cytometry. (B) Identification of exosomes by transmission electron microscopy. Scale bar=100 nm. (C) Representative image showing the presence of CM-Dil-labeled exosomes after co-culture with NSCs. Scale bar=200 μm. (D, E) The efficiency of Ndel1 overexpression was evaluated by RT-qPCR and Western blot. (F, G) The efficiency of Ndel1 knockdown was evaluated by RT-qPCR and Western blot. (H) The expression of miR-107-3p was evaluated by RT-qPCR. (I) Representative images of the hippocampus following lentivirus injection. Scale bar=400 μm. Values are the mean ± SEM from three biological replicates; ^*^P< 0.05, ^**^P< 0.01, ^***^P< 0.001.

**Additional file 2: Table S1.** The sequences of primers used for qRT-PCR

**Additional file 3: Table S2.** Differentially expressed mRNAs upregulated and downregulated in hippocampal exosomes

## Data Availability

Not applicable.

## Abbreviations

AD: Alzheimer’s disease
ALS: Amyotrophic lateral sclerosis
bFGF: Basic fibroblast growth factor 2
CNS: Central nervous system
CTE: Chronic traumatic encephalopathy
DG: Dentate gyrus
DMEM: Dulbecco’s modified Eagle’s medium
EGF: Epidermal growth factor
FF: Fimbria fornix
GO: Gene ontology
Ndel1: nudE neurodevelopment protein 1-like 1
HD: Huntington’s disease
NIH: National Institutes of Health
NSCs: Neural stem cells
PD: Parkinson’s disease
PBS: Phosphate-buffered saline
