# Supplementary figures and images for: Expression and function of Ndel1 during the differentiation of neural stem cells induced by hippocampal exosomesticle
Source: Stem Cell Res Ther. 2021 Jan 9;12:51. doi: 10.1186/s13287-020-02119-2 (PMC7796549; doi:10.1186/s13287-020-02119-2)

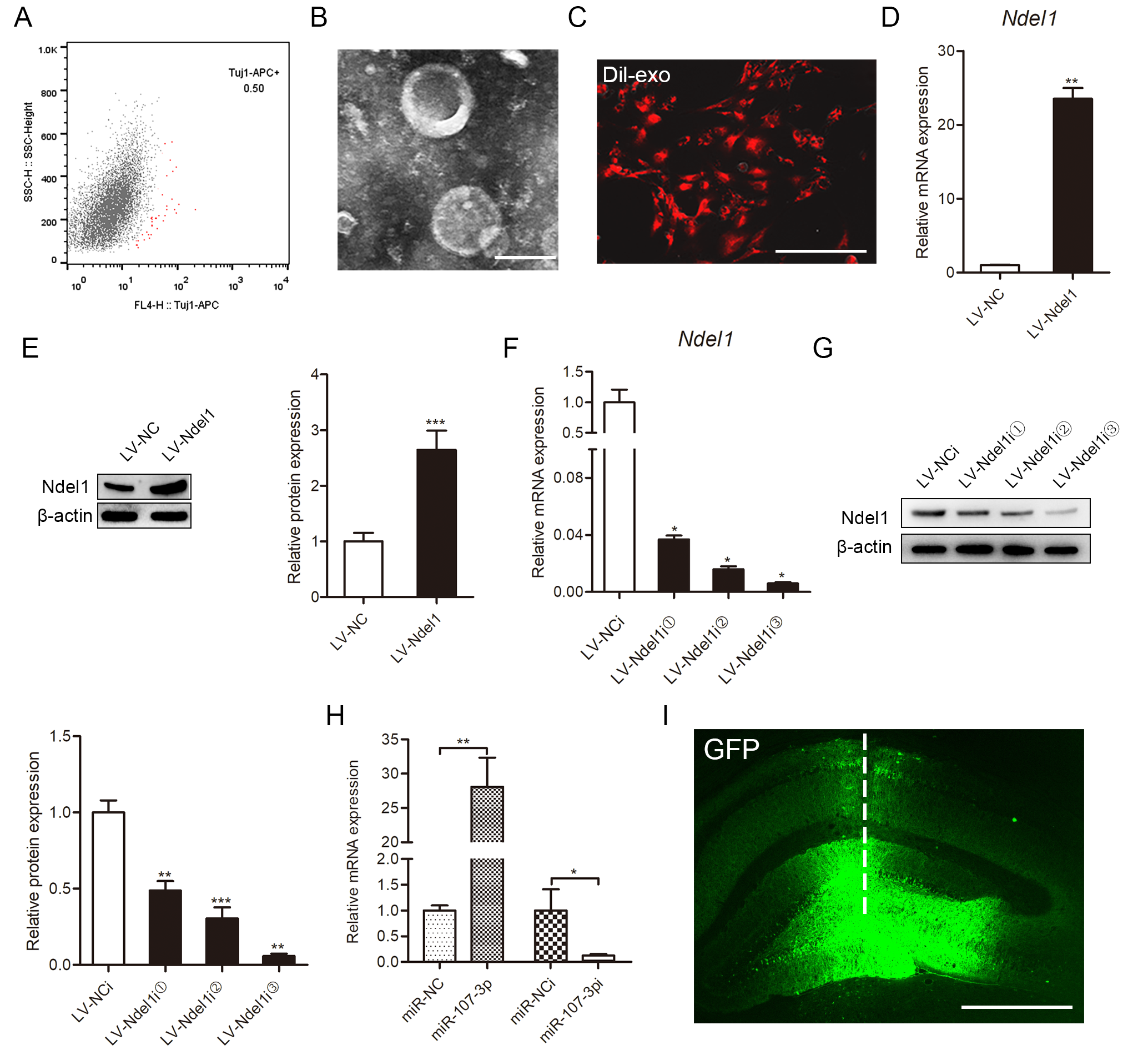

Supplement: Supplementary file 1 — Additional file 1: Figure S1. Representative images and histograms. (A) The control of flow cytometry. (B) Identification of exosomes by transmission electron microscopy. Scale bar=100 nm. (C) Representative image showing the presence of CM-Dil-labeled exosomes after co-culture with NSCs. Scale bar=200 μm. (D, E) The efficiency of Ndel1 overexpression was evaluated by RT-qPCR and Western blot. (F, G) The efficiency of Ndel1 knockdown was evaluated by RT-qPCR and Western blot. (H) The expression of miR-107-3p was evaluated by RT-qPCR. (I) Representative images of the hippocampus following lentivirus injection. Scale bar=400 μm. Values are the mean ± SEM from three biological replicates; *P< 0.05, **P< 0.01, ***P< 0.001. [file 13287_2020_2119_MOESM1_ESM.tif]
